# The relationship between histological prostatitis and lower urinary tract symptoms and sexual function

**DOI:** 10.1590/S1677-5538.IBJU.2015.0254

**Published:** 2016

**Authors:** Sukru Kumsar, Osman Kose, Huseyin Aydemir, Fikret Halis, Ahmet Gokce, Oztug Adsan, Zeynep Kahyaoglu Akkaya

**Affiliations:** 1 Sakarya University Training and Research Hospital, Sakarya, Turkey

**Keywords:** Lower Urinary Tract Symptoms, Prostatitis, Erectile Dysfunction

## Abstract

This prospective analysis assessed the effect of histological prostatitis on lower urinary tract functions and sexual function. The patients were separated into two groups as histologically observed prostatitis (Group A) and no prostatitis (Group B) according to the biopsy outcomes. International prostate symptom score, international index of erectile function-5 scores, maximal and average flow rate, and residual urine volumes were compared statistically between groups. There was no significant difference (P>0.05) in baseline age (t=0.64), body mass index value (t=0.51), prostate volume (t=0.87), prostate-specific antigen levels (t=0.43), maximal (t=0.84) and average flow rate (t=0.59), and post-void residual urine volume (t=0.71). Mean international prostate symptom score in patients with prostatitis was numerically but not significantly higher than that in those without prostatitis (t=0.794, P=0.066). Mean international index of erectile function-5 score in the prostatitis group was significantly lower than that in those without prostatitis (t=1.854, P=0.013). Histological prostatitis notably affected sexual function of patients and may serve as a major risk factor for sexual dysfunction while having little effect on lower urinary tract symptoms.

## INTRODUCTION

Histological prostatitis corresponds to prostatic inflammation confirmed by microscopic examination. The typical histologic finding of prostatitis is characterised by infiltration of the prostatic ductus and periprostatic tissue, especially with polymorphic nuclear leukocytes (1, 2).

Histological prostatitis is frequently detected in biopsy of prostate specimens during surgery or autopsy. A prostate autopsy study found inflammation in 40 of 91 adults patients (3).

Maksem et al. have reported evidence of prostatic inflammation in 45% of aspiration biopsy specimens taken because of suspicion of carcinoma (4).

Even though inflammatory cells in prostate tissue are a well-reported observation, there is no precise information about the origin of inflammation, which is thought to be multifactorial (5).

A number of factors, such as bacterial infection; chemical inflammation caused by urinary reflux, dietary factors, and hormones; and autoimmune responses, have been implicated in the development of prostatitis (6-8). Further, prostatic inflammation has an apparent correlation with symptomatic progression, risk of urinary retention, and need for surgery (9).

Recent studies have introduced data that prostatic inflammation plays an important role in the development and progression of benign prostatic hyperplasia (BPH) (10, 11).

Lower urinary tract symptoms (LUTS) caused by BPH impair quality of life and are frequently accompanied by sexual function disorders in these patients (12). Even though the mechanism underlying the relationship between BPH and erectile dysfunction (ED) is not exactly known, decrease in nitric oxide, metabolic syndrome, atherosclerosis, and the increase in Rho-kinase activity have been suggested as causes (13).

While prostatic inflammation is thought to play an active role in the progression of LUTS caused by BPH, no precise information exists on the relationship of this condition and sexual function disorders. The purpose of this study was to assess the effect of histological prostatitis on LUTS and sexual dysfunction.

## PATIENTS AND METHODS

**Figure 1:**
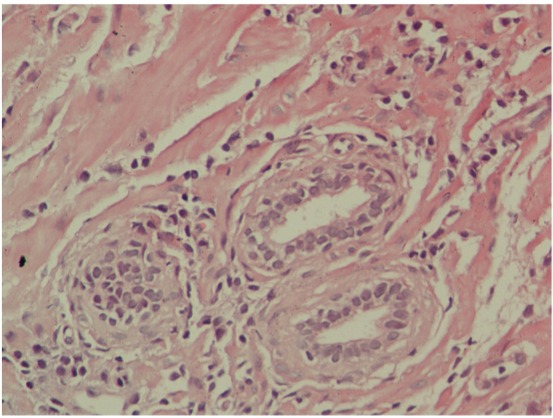
H&Ex400: This is the microscopic appearance of chronic prostatitis. Numerous small dark blue lymphocytes are seen in the stroma between the glands.

**Figure 2:**
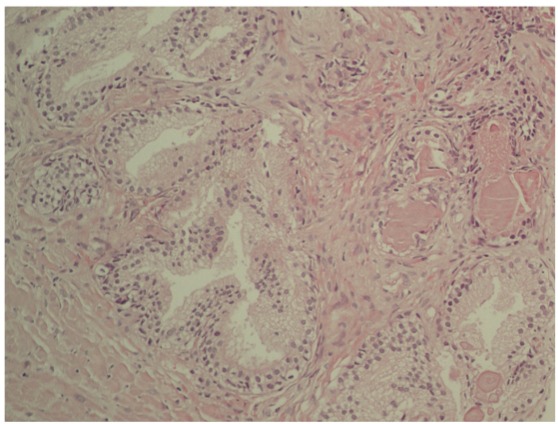
H&E: The normal histologic appearance of prostate glands and surrounding fibromuscular stroma is shown here at high magnification.

In total, 138 patients with serum PSA (ng/mL) above 4 with a normal digital rectal exam (DRE), and who were scheduled for transrectal prostate biopsy were included in the study. To evaluate lower urinary tract and sexual functions, international prostate symptom score (IPSS) and international index of erectile function-5 (IIEF-5) questionnaires were completed by patients before biopsy, respectively. Pre-biopsy uroflowmetry results (maximal flow rate [Qmax] and average flow rate [Qavg]) and post-void residual urine volume (PVR), as well as body mass index (BMI) and prostate volumes measured through transrectal ultrasonography, were recorded. Patients who received BPH or prostatitis treatment; those who were diagnosed with prostate cancer or atypia according to pathologic result; and those who had serious neurologic, cardiac, or pulmonary disorders, liver or renal failure, diabetes, or hypertension were excluded from the study. Prostatitis was diagnosed after histological observation of inflammatory cellular infiltration within the prostatic glandular tissue. Enrolled patients were divided into two groups: those with histological findings of prostatitis (Group A) (Figure-1) and those without prostatitis (Group B) (Figure-2) findings in their biopsy samples. IPSS and IIEF scores, as well as their uroflowmetry and residual urine volumes were compared statistically.

### Statistical analysis

SPSS software (Statistical Package for the Social Sciences; Version 15.0, SPSS Inc., Chicago, IL, USA) was used to analyse the data. The Kolmogorov–Smirnov test was used to evaluate whether the obtained data had a normal distribution. Normally distributed variables were described using means and standard deviations. Paired t-test was used to compare groups, and a P value<0.05 indicated statistical significance.

Independent (unpaired) samples t-test was used to determine whether there was any difference between groups in age, BMI, PSA, prostate volume, uroflowmetry parameters, and mean IPSS and IIEF scores, which are continuous variables. A chi-square test was used to compare smoking status, IPSS and IIEF severity.

A multivariate regression analysis was used to calculate the adjusted means of IPSS and IIEF score. Age, BMI, smoking status, PSA value, and prostate volume were used as continuous variables and as covariates in the model.

## RESULTS

Among the 138 patients in whom transrectal prostate needle biopsy was performed due to high PSA levels, 34 were excluded from the study: 28 had prostatic adenocarcinoma, and 6 had prostatic intraepithelial neoplasia (PIN). Of patients included in the study (n=104), 34.6% had histological prostatitis (Group A) and 65.4% had none (Group B).

There was no significant difference in age, BMI, prostate volume, PSA, Qmax, Qavg and PVR (Table-1). There was also no significant difference in prevalence of smoking (χ^2^=2.21).

**Table 1:** Comparison of the demographic characteristics of groups

	Group A	Group B	t value	P value
Age	63.9±5.9	64.1±6.8	0.643	0.080
BMI	26.0±2.5	26.5±3.4	0.512	0.320
Prostate volume	40.1±13.4	44.1±12.7	0.876	0.145
PSA	7.9±3.5	7.3±3.6	0.431	0.413
IPSS	19±7.4	17.6±8.5	0.794	0.066
IIEF	16.5±6.6	19.6±3.9	1.854	0.013
Qmax	11.3±5.2	10.1±4.8	0.841	0.236
Qavg	5.9±2.6	5.6±2.6	0.595	0.676
PVR	43.6±30.9	48.6±31.2	0.710	0.431

**Group A** = Histological prostatitis group; **Group B** = Without prostatitis group; P < 0.05; t-test for paired sample

In Group A, 15.6% had mild, 43.1% had moderate, and 41.3% had severe symptoms according to the IPSS. In contrast, in Group B, 17.6% had mild, 35.3% had moderate, and 47.1% had severe symptoms. There was no significant difference between groups according to IPSS severity (Table-2). Mean IPSS scores were 19±7.4 in Group A and 17.6±8.5 in Group B. Even though mean IPSS in Group A was numerically higher than that in Group B, this result was not statistically significant (t=0.794, P=0.066) (Table-1).

**Table 2:** Comparison of IPSS severity between the two groups (chi-square test).

IPSS severety	Group A (%)	Group B (%)	P value
Mild	15.6	17.6	0.085
Moderate	43.1	35.3	0.135
Severe	41.3	47.1	0.068

**Group A** = Histological prostatitis group; **Group B** = Without prostatitis group

ED was mild in 33.3%, moderate in 16.7%, and severe in 19.4% patients and 30.6% did not have ED in Group A according to the IIEF scores. In Group B, ED was reported as mild in 35.3% and moderate in 23.5% patients and 41.2% did not have ED. According to these results, severe ED was significantly high in group A (Table-3).

**Table 3:** Comparison of IIEF severity between the two groups (chi-square test).

IIEF severety	Group A (%)	Group B (%)	P value
No ED	30.6	41.2	0.126
Mild	33.3	35.3	0.085
Moderate	16.7	23.5	0.062
Severe	19.4	0	<0.001

**Group A:** Histological prostatitis group; **Group B:** Without prostatitis group

Mean IIEF scores were 16.5±6.6 in Group A and 19.6±3.9 in Group B. The mean IIEF score was significantly high in Group A (t=1.854, P=0.013) (Table-1). The multivariate regression analysis found that age, PSA, prostate volume, BMI, and smoking had no effect on IIEF scores. Age, PSA, BMI, and smoking had no effect on IPSS; however, prostate volume correlated with IPSS.

## DISCUSSION

Our study found that patients diagnosed with histological prostatitis had more serious erectile dysfunction than those without prostatitis.

Recent studies have frequently focused on the effects of histologic inflammation of prostate tissue on progression of BPH, LUTS, and sexual function. With an extensive and extended follow-up, the Medical Treatment of Prostate Symptoms (MTOPS) study found prostatic inflammation in 544 of 1197 patients with BPH. A correlation was observed between histological prostatitis and clinical progression of BPH. Patients in all groups (placebo, finasteride, doxazosin, and combined finasteride and doxazosin) with inflammation were more likely to progress clinically in terms of symptoms, acute urinary retention (AUR), or BPH-related surgery (14).

Another study on the pathophysiology between BPH and prostatitis theorised that cytokines released from the inflammatory cells in prostate tissue and growth factors stimulated epithelial and stromal hyperproliferation (11).

In addition, cellular proliferation repaired the tissue damage caused by the free oxygen radicals arising from the released cytokines caused hypoxia (15).

The Reduction by Dutasteride of Prostate Cancer Event (REDUCE) study observed chronic inflammation on histological analysis in 78% men. Statistically significant but clinically small increases in IPSS were noted in patients with inflammation compared with those without inflammation. Similarly, statistically significant correlations were found between average chronic inflammation score and the IPSS variables (16).

In a cohort study of 282 patients, Robert et al. observed a significant correlation between the degree of prostatic inflammation, prostate volume, and urinary system symptoms. Mean IPSS was 12 in patients with lower inflammation and 21 in patients with higher inflammation (17).

In contrast, our study grouped patients by the existence of prostatitis; 34.6% patients had prostatic inflammation. Mean IPSS was 19 in patients with prostatitis and 17.6 in patients without prostatitis. The difference was not statistically significant. Similarly, Edlin et al. in their study evaluating the prevalence of prostatic inflammation in BPH and prostatic adenocarcinoma, reported histological prostatitis in 61% patients with BPH and that histological prostatitis showed a minor correlation with LUTS (18).

Sexual function disorders are frequently encountered in patients with chronic prostatitis. Liang et al. observed 2000 patients with chronic prostatitis and found that 49% of patients had comorbid sexual dysfunction. Moreover, 26.4% of these patients had premature ejaculation, 14.9% erectile dysfunction, and 7.7% both conditions (19).

The effect of histological prostatic inflammation on ED is gaining importance recently. Abdullah et al. showed that upon reduction of postsurgical inflammation in patients with prostatitis and BPH, sexual function improved (20).

Wang et al. revealed that the presence of prostatitis in tissues of patients with BPH undergoing a transurethral prostate resection had a serious effect on the sexual function and a less effect on LUTS (21).

## CONCLUSIONS

In our study, among patients in whom biopsy was performed, IIEF scores were significantly lower in those with inflammation at the tissue level than in those without prostatitis. We suggest that the mechanism underlying advances in tissue damage caused by inflammation and BPH may also affect sexual function with similar mechanisms. Additional clinically relevant study is needed.
